# The COVID-19 Pandemic: How Technology Is Reshaping Public Health and Medicine

**DOI:** 10.3390/bioengineering10050611

**Published:** 2023-05-19

**Authors:** Luís Coelho, Dimitrios Glotsos, Sara Reis

**Affiliations:** 1 ISEP—School of Engineering, Polytechnic Institute of Porto, 4200-465 Porto, Portugal; 2 INESCTEC, Campus da Faculdade de Engenharia da Universidade do Porto, 4200-465 Porto, Portugal; 3 Biomedical Engineering Department, University of West Attica, 122 43 Athens, Greece; dimglo@uniwa.gr; 4 CIETI, Polytechnic Institute of Porto, 4200-465 Porto, Portugal; ssr@isep.ipp.pt

The outbreak of the novel coronavirus severe acute respiratory syndrome coronavirus 2 (SARS-CoV-2) pandemic has been a watershed moment in human history, causing a profound shift in the global landscape that has affected every aspect of our lives. The virus has not only brought the world to a standstill but has also challenged our values, beliefs, and societal structures, exposing the vulnerabilities and inequalities that exist within our systems.

The COVID-19 pandemic has posed unprecedented challenges to the global community, and technology has emerged as a key tool in managing the spread of the virus and mitigating its impact on society. From contact tracing to remote work and telemedicine, technology has enabled us to respond to the crisis in innovative and effective ways.

Bioengineering is a multidisciplinary field that applies engineering principles and techniques to solve problems in the life sciences. This field is characterized by its broad scope and diverse applications, which range from designing new medical devices to developing innovative therapies for various diseases. Bioengineering is concerned with understanding biological systems, from the molecular level to the organ and organismal level, and using this knowledge to develop technologies and therapies that can improve human health and well-being.

Additionally, artificial intelligence (AI) tools have emerged as a critical component of bioengineering, offering significant potential to revolutionize the field. With the ability to analyze vast amounts of data, AI has become an indispensable tool in the development of new treatments, drugs, and medical technologies. AI can be used to develop predictive models that simulate complex biological processes, identify potential therapeutic approaches, and optimize experimental design. Machine learning algorithms are also used to extract insights from large data sets, helping researchers to uncover new connections and relationships that would be difficult to detect using traditional methods. Additionally, AI-enabled technologies such as robotics and computer vision have made it possible to automate many laboratory processes, allowing researchers to conduct experiments more efficiently and with greater accuracy.

AI also plays a critical role in the development of new therapies for a variety of diseases, including cancer, cardiovascular disease, and neurological disorders. By applying engineering principles and techniques to the study of these diseases, bioengineers can develop new therapies that target specific molecular pathways, identify new biomarkers for disease diagnosis and monitoring, and design more effective clinical trials to evaluate the safety and efficacy of new treatments.

In fact, disease detection and the effective diagnosis of its severity were crucial for a good prognosis in the context of COVID-19. In Chadaga et al. [1], the authors proposed a decision support system that uses machine learning and deep learning techniques to predict the COVID-19 diagnosis of a patient using clinical, demographic, and blood markers. The patient data used in this research were collected from two Manipal hospitals in India and a custom-made, stacked, multi-level ensemble classifier has been used to predict the COVID-19 diagnosis. Deep learning techniques such as deep neural networks (DNN) and one-dimensional convolutional networks (1D-CNN) were also utilized. Furthermore, explainable artificial techniques (XAI) such as Shapley additive values (SHAP) [2], ELI5 [3], local interpretable model explainer (LIME) [4], and QLattice [5,6] were used to make the models more precise and understandable. Among all of the algorithms, the multi-level stacked model obtained an excellent accuracy of 96%. The precision, recall, f1-score, and AUC obtained were 94%, 95%, 94%, and 98% respectively. The proposed classifiers can be used as a decision support tool for healthcare professionals, allowing faster detection of the disease.

The use of XAI algorithms is paramount in healthcare-related areas. They enable us to identify and correct errors or biases in the models allowing us to build trust in the predictions made by AI systems. In healthcare, where the stakes are high, explainability is crucial for ensuring that AI systems are making accurate and fair predictions. Additionally, these algorithms can bring further insight into complex and large-dimensional problems as well as their related prediction models. XAI algorithms, such as decision trees and rule-based models, can help to explain the predictions made by these models by providing a transparent and interpretable framework for decision-making. This information can help clinicians to understand the factors that are driving the predictions and make more informed treatment decisions.

The same algorithms can be applied to images (e.g., ultrasound, X-Rays, or MRIs), an area where deep learning models are increasingly being used. These models, due to their intricate architectures, are often difficult to interpret, and it can be challenging to understand how they arrived at their diagnoses. Algorithms such as SHAP and LIME can help to explain the predictions made by these models by highlighting the regions of the image that were most important in making the diagnosis. This information can help radiologists to validate the model’s predictions and improve the accuracy of the diagnosis.

Medical imaging has played a critical role in the diagnosis and management of COVID-19. Chest X-ray radiographs and X-ray computed tomography (X-ray CT) scans have been used to identify characteristic features of the disease such as ground-glass opacities and consolidations in the lungs. These imaging modalities have been particularly useful in the early detection of the disease and have helped to guide patient management and treatment decisions. However, the interpretation of medical images, as mentioned, can be challenging, and the accuracy of the diagnosis depends on the skill and experience of the radiologist. As such, machine learning algorithms are being developed to assist with the diagnosis. Gazzoni et al. [7] explored the advantages of using Lung Ultrasound (LUS) over chest X-rays (CXR) for this purpose. The supporting dataset contains SARS-CoV-2 patterns, ranked according to three severity scales, comprising ultrasounds from linear and convex probes in 5400 clips from 450 hospitalized subjects. A standardized severity ranking scale to evaluate pneumonia was used. A video-classification approach based on contemporary DL strategies was developed in close collaboration with Fondazione IRCCS Policlinico San Matteo’s Emergency Department (ED) of Pavia. The authors proposed a new method that combines an X3D [8] network architecture with key-frame selection to classify ultrasound videos as positive or negative cases for SARS-CoV-2. The proposed method achieved high accuracy, sensitivity, and specificity, with an overall accuracy of 93.8%, a sensitivity of 93.7%, and a specificity of 93.8%. This research has several significant implications for the medical community. First, it provides an alternative diagnostic tool for COVID-19 that can be used in situations where other methods are not available or not feasible. Second, it highlights the potential of deep learning techniques in the field of medical imaging, particularly for the analysis of ultrasound videos.

It is important to highlight that the application of classification algorithms to medical videos is not limited to a single modality. These techniques have been widely utilized across various medical imaging modalities, including chest X-rays. In light of the COVID-19 pandemic, the scientific community has come together to address this global health crisis by providing access to large datasets of chest X-rays for the detection of the virus [9,10,11,12,13]. This has led to a surge of interest in applying classification algorithms to chest X-ray images, which has the potential to significantly impact the diagnosis and treatment of COVID-19. The rapid availability of these datasets has enabled researchers to develop and validate AI-based models for the accurate and efficient detection of COVID-19, which was crucial in managing the spread of the disease [14,15]. Thus, the surge of the pandemic has contributed to a fast development of high-performance classification algorithms in medical imaging that has far-reaching implications for the future of healthcare. Nevertheless, there are some concerns about the quality of the available datasets since they can contain a disproportionate number of X-rays from certain populations, such as older patients or those with more severe symptoms [16]. Bias in datasets used to train AI models can lead to inaccurate diagnoses and negatively impact patient outcomes. Therefore, it is imperative to prioritize the development of more diverse and representative datasets to ensure the accuracy and reliability of AI models. This is particularly important in the context of COVID-19, where timely and accurate diagnoses can save lives and prevent the spread of the disease. As such, it is essential that future developments in AI-based COVID-19 diagnosis and management take into account the need for unbiased and representative datasets, and that researchers work towards creating inclusive and equitable datasets to improve the quality of care for all patients.

Still concerning diagnosis, the use of speech, breathing, and cough must be mentioned [17,18]. These modalities offer the possibility of low-cost non-invasive mass self-screening that allows a fast diagnosis. These tools can be essential in the prevention and management of epidemic outbreaks with a high degree of contagion.

Besides diagnostic tools, it is also important to understand risk factors and disease evolution patterns to properly prepare healthcare services for peak demands. The use of big data and machine learning has also enabled public health officials to identify risk groups, model the spread of the virus, predict hotspots, and plan resource allocation more effectively. These are crucial for public health planning and management and also for healthcare providers.

Colnago et al. [19] performed a retrospective analysis of medical records of patients with confirmed COVID-19 who were hospitalized between December 2021 and February 2022. The authors identified age, obesity, chronic kidney disease, chronic obstructive pulmonary disease, and the use of mechanical ventilation as the main risk factors associated with mortality. The study underscores the importance of early identification and management of these risk factors in reducing mortality in hospitalized patients with COVID-19 during the Omicron wave. The findings could help guide the development of targeted interventions and personalized treatment strategies for COVID-19 patients in Brazil and beyond.

Moreover, Carvalho et al. [20] analyzed and forecasted the incidence, in intensive care unit admissions, and projected mortality attributable to COVID-19 in Portugal, the UK, Germany, Italy, and France, predicting for 4 weeks ahead, giving the opportunity to prepare the healthcare system with the necessary resources. The presented study is based on publicly available data and used machine learning techniques to develop predictive models that take into account a range of factors, including demographics, population density, climate, and social distancing measures. Overall, the paper highlighted the potential of machine learning and data analytics in predicting and managing the spread of COVID-19. By providing accurate and timely forecasts of COVID-19 incidence, ICU admissions, and mortality, these models can help governments and healthcare providers to allocate resources more effectively, plan for future outbreaks, and develop targeted interventions to mitigate the impact of the pandemic on public health and the economy.

Finally, technology has also played a critical role in facilitating the development and distribution of vaccines. Scientists have used advanced computational models and artificial intelligence to accelerate the development of vaccines in a record time. In [21], Zhang et al. presented a comprehensive overview of the current state of research on advanced vaccine design strategies for SARS-CoV-2 and its emerging variants. The authors provided a detailed analysis of the various types of vaccines currently available, including mRNA vaccines, viral vector vaccines, protein subunit vaccines, and inactivated vaccines, and their efficacy against different variants of the virus. The paper also highlighted the importance of personalized medicine in vaccine development and distribution. The authors argued that personalized medicine approaches, such as the use of individualized vaccine dosing and the incorporation of patient-specific data into vaccine design, could significantly improve vaccine efficacy and reduce adverse reactions. The proposed strategies have the potential to significantly improve vaccine efficacy, address emerging challenges posed by new variants of the virus, and ultimately contribute to the control and management of the COVID-19 pandemic.

Additionally, bioengineering has also been instrumental in the development of new therapies for COVID-19, from which we can emphasize the use of convalescent plasma and monoclonal antibodies, which have been shown to be effective in reducing the severity of the disease in some patients [22,23].

In summary, the COVID-19 pandemic has demonstrated the power of technology in addressing complex challenges and helping to mitigate the impact of the virus on society. As we emerge from the pandemic, it is clear that technology will continue to play a critical role in shaping our response to future crises, enabling us to work more efficiently, connect more effectively, and build a more resilient and sustainable future. The integration of AI tools and bioengineering has the potential to further accelerate the pace of innovation in healthcare and improve patient outcomes.

We are confident that this Special Issue will serve as a valuable resource for our readers, providing them with a fresh perspective on how bioengineering and machine learning were used to combat the pandemic and this was an opportunity to boost scientific knowledge. We would like to express our heartfelt gratitude to the many dedicated reviewers who have worked tirelessly to make this issue possible. Their unwavering commitment, invaluable insights, and diligent efforts have been instrumental in shaping this Special Issue. Without their selfless contributions, this issue would not have been possible. We are honored to have had the opportunity to collaborate with such a dedicated and talented group of individuals, and we look forward to future collaborations that will further advance our understanding and application of emerging technologies in the fight against pandemics.
